# Association of Long Non-Coding RNA Growth Arrest-Specific 5 Genetic Variants with Diabetic Retinopathy

**DOI:** 10.3390/genes13040584

**Published:** 2022-03-25

**Authors:** Chee-Ming Lee, Yi-Sun Yang, Edy Kornelius, Chien-Ning Huang, Min-Yen Hsu, Chia-Yi Lee, Shu-Yen Peng, Shun-Fa Yang

**Affiliations:** 1 Institute of Medicine, Chung Shan Medical University, Taichung 402, Taiwan; jen.y287@mail.jah.org.tw (C.-M.L.); cshy049@gmail.com (C.-N.H.); pengshuyen@gmail.com (S.-Y.P.); 2 Department of Ophthalmology, Jen-Ai Hospital, Taichung 412, Taiwan; 3 School of Medicine, Chung Shan Medical University, Taichung 402, Taiwan; monica119@gmail.com (Y.-S.Y.); korn3lius82@gmail.com (E.K.); my.scott.hsu@gmail.com (M.-Y.H.); 4 Department of Internal Medicine, Division of Endocrinology and Metabolism, Chung Shan Medical University Hospital, Taichung 402, Taiwan; 5 Department of Ophthalmology, Chung Shan Medical University Hospital, Taichung 402, Taiwan; 6 Department of Ophthalmology, Nobel Eye Institute, Taipei 115, Taiwan; ao6u.3msn@hotmail.com; 7 Department of Medical Research, Chung Shan Medical University Hospital, Taichung 402, Taiwan

**Keywords:** growth arrest-specific 5, polymorphism, diabetic retinopathy, diabetes mellitus, duration

## Abstract

The aim of this work was to appraise the potential associations of single nucleotide polymorphisms (SNPs) of long non-coding RNA growth arrest-specific 5 (*GAS5*) with diabetic retinopathy (DR) in a diabetes mellitus (DM) population. Two loci of the *GAS5* SNPs (rs55829688 and rs145204276) were genotyped via TaqMan allelic discrimination in 449 non-DR patients and 273 DR subjects. The SNP rs145204276 Del/Del showed a significantly higher distribution in the DR group compared to the non-DR group (AOR: 2.487, 95% CI: 1.424–4.344, *p* = 0.001). During subgroup analyses, the non-proliferative diabetic retinopathy (NPDR) subgroup demonstrated a significantly higher ratio of the SNP rs145204276 Del/Del (AOR: 2.917, 95% CI: 1.574–5.406, *p* = 0.001) and Ins/Del + Del/Del (AOR: 1.242, 95% CI: 1.016–1.519, *p* = 0.034) compared to the non-DR population, while the proliferative diabetic retinopathy (PDR) subgroup did not reveal significant differences in either SNP rs145204276 or rs55829688 distributions compared to the non-DR group. Furthermore, patients with a *GAS5* SNP rs145204276 Del/Del showed a significantly shorter DM duration than the wild type (Ins/Ins) (*p* = 0.021). In conclusion, our findings demonstrate that the *GAS5* SNP rs145204276 Del/Del variant is associated with an increased susceptibility to DR in DM patients, particularly in those patients with NPDR.

## 1. Introduction

Diabetes mellitus (DM) is a prevalent disease, affecting nearly 9% of adults worldwide according to a recent review [1]. The clinical features of DM include a hyperglycemia status with a high glycated hemoglobin (HbA1c) level [2], and some vascular disorders are also associated with DM. Diabetic retinopathy (DR) is a DM-related complication in the eye due to ocular blood vessel damage [3], which can be divided into non-proliferative diabetic retinopathy (NPDR) and proliferative diabetic retinopathy (PDR) based on the presence or absence of retinal neovascularization [4]. In advanced stages, DR may cause the induction of neovascular glaucoma and other disorders that could lead to permanent vision loss [4,5].

Several factors are correlated with the formation or progression of DR [3,6,7]. The duration of DM and the condition of blood sugar control are fundamental risk factors for the development of DR [7]. In addition, inflammatory cytokines, oxidative stress, growth factors including the vascular endothelial growth factor, and adhesion molecules were proven to be associated with DR [6]. Regarding the genetic aspect of DR, a single nucleotide polymorphism (SNP) of interleukin-10 (rs11567245, A > G) was found to be related to DR clinical indication [8]. The SNP rs149465 (T > A) of aquaporin 4 and the SNP rs11984041 (C > T) of histone deacetylase 9 have also been associated with the development of DR [9,10]. Furthermore, the SNP rs1617640 (T > G) of erythropoietin has been found to increase the risk of PDR [11], implying that genetic polymorphisms may produce different effects at different DR stages. Long-non-coding RNAs are a group of transcripts that each contain more than 200 nucleotides but are not translated into proteins, and are participants in transcriptional regulation, nuclear domain regulation, and cancer development [12,13,14,15,16,17]. For instance, the SNP rs527616 (C > G) of the long-non-coding RNA AQP4-AS1 has been found to increase susceptibility to breast cancer [18], and the SNP rs564398 (C > T) of the long-non-coding RNA ANRIL is associated with both coronary artery disease and type 2 DM [19,20]. Accordingly, it is possible that certain long-non-coding RNAs are related to DM or DM-related complications such as DR.

The growth arrest-specific 5 (*GAS5*) RNA, a long non-coding RNA, has been proven to alter the apoptosis of breast cancer cells, the angiogenesis of colorectal cancer, the tumorigenesis of non-small cell lung cancers, and the expression of insulin receptors in the previous literature [21,22]. Recently, the SNPs of *GAS5* were found to be associated with the clinical characteristics of oral cancer and prostate cancer [23,24]. Regarding the potential relationship between *GAS5* and DR, one study demonstrated that the expression of circulating *GAS5* was significantly reduced in DM patients but there was no significant difference in *GAS5* expression between DR and non-DR individuals [25]. Nevertheless, the genetic polymorphism of *GAS5* was not evaluated in that study. Since the modification of *GAS5* promotes capillary leakage and neovascularization [26], the SNPs of *GAS5* may also affect the development or progression of DR via impairing the vasculature, although this needs validation.

The purpose of this study was to appraise the potential effect of *GAS5* SNPs on the clinical characteristics of DR in a Taiwanese population. Furthermore, the effects of *GAS5* SNPs on NPDR and PDR were analyzed separately in subgroup analyses.

## 2. Materials and Methods

### 2.1. Ethic Declarations

All procedures in this study adhered to the Declaration of Helsinki from 1964 and its later amendments. In more detail, our study was approved by the Institutional Review Board of Chung Shan Medical University Hospital (project identification code: CS1-20048). Signed written informed consent forms were obtained from all subjects participating in this study, which are available upon reasonable request.

### 2.2. Subject Selection

The prospective case-control study was administered in the Chung Shan Medical University Hospital. A total number of 722 individuals diagnosed with DM were included in this study. Of these, 449 patients were designated into the non-DR group and another 273 individuals into the DR group in accordance with the medical records of the ophthalmic department. The presence of DR was defined as one of the following fundus findings in the ophthalmic records: dot/flame shape hemorrhage, hard exudate, cotton-wool spot, microaneurysm, venous beading, or intraretinal microvascular abnormality. Additionally, among the 273 patients in the DR group, 110 participants were categorized into the PDR group because one of the subsequent findings was found in the ophthalmic records: neovascularization of optic disc or retina, vitreous hemorrhage, tractional retinal detachment, or neovascular glaucoma. The remaining 163 participants in the DR group were regarded as the NPDR group. The ophthalmic documents were recorded by four experienced ophthalmologists.

### 2.3. Data and Sample Collection

The medical documents of these participants at Chung Shan Medical University Hospital were checked, and related data including age, sex, body mass index, blood pressure, lipid profiles, blood sugar level presented as HbA1c, duration of DM, presence of insulin treatment, and renal function were recorded. For the assessment and analyses of *GAS5* polymorphism, venous-blood-drawing was performed in every participant at the Chung Shan Medical University Hospital, and these venous blood samples were stored in ethylenediaminetetraacetic acid-containing tubes. Then, these samples were centrifuged immediately and preserved in one laboratory refrigerator maintained at approximately −80 °C. Of note, patients that provided venous blood samples were excluded from this study if the genomes of the venous blood sample were degraded before genetic analyses.

### 2.4. DNA Extraction and Determination of GAS5 SNP via Real-Time PCR

Two SNPs of *GAS5*, rs145204276 (Ins/Del) and rs55829688 (T/C), were chosen for analysis because the minor allele frequencies of both SNPs are more than 5% and our earlier studies implied an effect on malignancy [23]. The gene extraction procedures in this study were similar to those in our previous research [27,28]. Firstly, genomic DNA was extracted from leukocytes in these venous blood samples via the use of QIAamp DNA kits (Qiagen, Valencia, CA, USA). We undertook this procedure according to the manufacturer’s instructions for DNA isolation. We dissolved DNA in Tris-EDTA (TE) buffer (10 mM Tris and 1 mM EDTA; pH 7.8) and quantified it by measuring the optical density at 260 nm and a A260/A280 ratio. Then, isolated DNA samples were preserved at around −20 °C. The genetic polymorphism concerning both *GAS5* SNPs, rs145204276 and rs55829688 (T/C), was determined with an ABI StepOne Real-Time PCR System (Applied Biosystems, Foster City, CA, USA). The final results of *GAS5* genetic polymorphism were analyzed via SDS version 3.0 software (Applied Biosystems, Foster City, CA, USA). 

### 2.5. Statistical Analysis

SAS version 9.4 (SAS Institute Inc., Cary, NC, USA) was applied for the statistical analyses in our study. The descriptive analysis, including mean, standard deviation (SD), and percentage data, was used to show the demography and laboratory data between the non-DR and DR groups. Independent tests were applied to compare the differences in the demography and laboratory data between the non-DR and DR groups. Then, multiple logistic regression models were adopted to estimate the adjusted odds ratio (AOR) and corresponding 95% confidence intervals (CI) of the SNPs’ distribution between the non-DR and DR groups, adjusting for the effects of age, the duration of diabetes, HbA1c, insulin treatment, serum creatinine levels, glomerular filtration rate, and HDL cholesterol levels. A similar pattern of multiple logistic regression models was used in the subgroup analyses regarding the SNP distribution in the NPDR and PDR subgroups compared to the non-DR group. In the next step, the differences in the clinical characteristics among each SNP rs145204276 phenotype (i.e., the Ins/Ins, Ins/Del, and Del/Del) in the DR group were analyzed via a one-way analysis of variance with Tukey’s a posteriori comparison. A *p* value less than 0.05 was regarded as statistically significant in this study.

## 3. Results

### 3.1. Characteristics between the Non-DR and DR Groups

The basic characteristics of the non-DR and DR groups are shown in Table 1. The mean age was 62.57 ± 10.77 in the DR group, which was significantly higher than the non-DR group (60.20 ± 11.24, *p* = 0.005). In addition, the diabetic parameters including the duration of DM, HbA1c level, and percentage of insulin treatment were all worse in the DR group (all *p* < 0.001). Regarding the laboratory data, the DR group showed a more impaired renal function (both *p* < 0.001) and a lower HDL level (*p* = 0.013).

### 3.2. GAS5 SNPs Distribution among Different DR Groups

After adjusting for many demographic and laboratory factors, the SNP rs145204276 Del/Del demonstrated a significantly higher distribution in the DR group compared to the non-DR group (AOR: 2.487, 95% CI: 1.424–4.344, *p* = 0.001). The other distribution of the SNP rs145204276 and all distributions of the SNP rs55829688 did not reveal significant differences between the non-DR and DR groups (all *p* > 0.05) (Table 2). In the subgroup analyses, the NPDR subgroup illustrated a significantly higher ratio of SNP rs145204276 Del/Del (AOR: 2.917, 95% CI: 1.574–5.406, *p* = 0.001) and Ins/Del + Del/Del (AOR: 1.242, 95% CI: 1.016–1.519, *p* = 0.034) compared to the non-DR group. The other SNP distributions between the non-DR group and NPDR subgroup were similar (all *p* > 0.05) (Table 3). Regarding the PDR subgroup, all the distributions of both SNP rs145204276 and SNP rs55829688 did not reveal significant differences between the PDR subgroup and the non-DR group (all *p* > 0.05) (Table 4).

### 3.3. Clinical Characteristics and Distribution of GAS5 SNP rs145204276 in DR Patients

Concerning the clinicopathological characteristics of the DR population with different SNP rs145204276, participants with the *GAS5* SNP rs145204276 variant (Del/Del) demonstrated a significantly shorter duration of DM compared to the wild type (Ins/Ins) (*p* = 0.021). The other parameters such as HbA1c level, renal function, and lipid profiles revealed similar values among the three *GAS5* SNP rs145204276 types (all *p* > 0.05) (Table 5).

## 4. Discussion

Briefly, this study demonstrated the significantly higher ratio of *GAS5* SNP rs145204276 in patients with DR. Moreover, this phenomenon is more prominent in those diagnosed with NPDR. Besides, the DR patients with the *GAS5* SNP rs145204276 variant experienced a shorter DM duration compared to the DR patients with the wild type *GAS5* SNP rs145204276.

The development of DR in DM patients is affected by several biochemical factors [6]. Inflammatory cytokines including interleukins and tumor necrosis factors have been proven to be associated with the formation of DR [6]. In a previous study, the expression of interleukin-6 was significantly associated with disease progression in PDR [29]. Additionally, tumor necrosis factor-α was associated with DR development in another study [30]. In addition to inflammatory cytokines, VEGF plays a crucial role in the development of DR and especially PDR [6]. DR originates due to retinal ischemia and hypoxia, which lead to the release of VEGF [3]. When the high expression of VEGF persists for a period of time, retinal neovascularization and subsequent PDR can develop [31]. In the treatment of DR, intraocular injection of anti-VEGF is a widely applied modality with fair outcomes on neovascularization regression and vision preservation [3]. In the aspect of genetic polymorphism, the development of DR may be affected by renalase, erythropoietin, and VEGF whereby certain SNP variants of these genes are found with higher frequency in patients with DR [11,32,33]. Regarding *GAS5*, the lncRNA is involved in several forms of cellular proliferation, cell differentiation, cell transition, immune disorders, and angiogenesis [21,34,35,36,37]. In previous literature, the level of *GAS5* is decreased in various cancers including breast cancer and gastric cancer [37]. Besides which, lower *GAS5* expression is associated with tumor angiogenesis in lung cancer [38]. In addition to *GAS5* itself, the genetic polymorphism of *GAS5* can influence the development of cancer and related angiogenesis [39]. For instance, the *GAS5* SNP rs145204276 has been found to be significantly prevalent in those with glioma and oral cancer [22,23]. Because DR is related to high VEGF concentrations and angiogenesis, and *GAS5* may lead to the increment of angiogenesis [40], certain SNPs of *GAS5* may be expressed more frequently in DR. The above concept was supported by the results of this study, at least partially.

In this study, the *GAS5* SNP rs145204276 was significantly prevalent in those with DR compared to the non-DR participants. To our knowledge, this is a relatively novel finding that has rarely been reported elsewhere. Besides this, several demographic and laboratory factors were enrolled in the multivariable analysis; thus, the *GAS5* SNP rs145204276 may be an independent indicator of DR status. Concerning the relationship between *GAS5* and DR, previous literature has demonstrated an insignificant association between *GAS5* expression and DR development [25]. However, this study only measured the concentration of *GAS5* in the circulatory system without the evaluation of genetic polymorphism [25]. In preceding research, *GAS5* can induce or reduce angiogenesis in different clinical conditions [40,41]. Consequently, we speculate that wild type *GAS5* does not affect the occurrence of DR, but the mutated type of *GAS5* with the SNP rs145204276 variant might promote angiogenesis in the retina and thus DR may develop in such situations. In the subgroup analyses, the *GAS5* SNP rs145204276 variant was related to the presence of NPDR, while only an insignificant correlation was observed between the *GAS5* SNP rs145204276 variant and the existence of PDR. Because the development of PDR is more dependent on VEGF expression than that of NPDR [31], it may be that the solitary genetic polymorphism of *GAS5* cannot induce adequate angiogenesis effects for PDR development. Further studies are warranted to survey this concept.

Regarding the clinical characteristics among the different *GAS5* SNP rs145204276 types in the DR population, the DR patients with a Del/Del variant of *GAS5* SNP rs145204276 exhibited a significantly short duration of DM according to the multivariable analyses. The mean DM interval in the Del/Del variant subgroup was 9.46 years, which was approximately four years and two years shorter than the disease interval in the wild type and Ins/Del subgroups numerically. The short disease duration of DM in this subgroup may indicate that the development of DR would be faster compared to the DM population of wild type *GAS5* SNP rs145204276, even if the patients showed similar HbA1c levels. Although we cannot ensure that the patients developed DR after our examination and some participants could have developed DR months or years before visiting our hospital, the results in this study may reveal a tendency of early-onset DR in the *GAS5* SNP rs145204276 Del/Del variant whether receiving ophthalmic examination periodically or not. Interestingly, the *GAS5* SNP rs145204276 Del/Del variant is also correlated with poor-differentiation cell status and a worse tumor stage, as well as a larger tumor size in oral cancer based on our previous study [23]. This coincidence might indicate that the *GAS5* SNP rs145204276 variants, especially the Del/Del variant, possess universal influences on tumor progression and angiogenesis-related disorders in Asian populations.

When it comes to the demographic data, laboratory exams and treatments of DM between the non-DR and DR groups, the age in the DR group was significantly higher than the non-DR group. Because age is an established risk factor for both DM and DR development [1], it is reasonable that the DR group showed an older age compared to the non-DR counterpart. The duration of DM, HbA1c level, and percentage of insulin treatment were all higher in the DR group. This may demonstrate a poorer DM control in the DR group, which is a concluded predictor of DR development [3,7]. On the other hand, the renal function and HDL concentrations were worse in the DR group while the blood pressure and other lipid profiles in the DR group were not inferior to those in the non-DR group. The possible explanations for the worse renal function in the DR group may be both that poor DM control can damage the kidney [1], and that chronic kidney disease has been correlated with several ocular diseases in previous studies [42,43,44]. Still, the reasons for the lower HDL level in the DR group need additional study to clarify.

Some limitations existed in this study. Firstly, the case-control design in this study cannot evaluate the effect of *GAS5* SNP longitudinally compared to the cohort design. Secondly, the number of cases in the *GAS5* SNP rs145204276 Del/Del subgroup in the DR group was relatively low compared to the Ins/Ins and Ins/Del *GAS5* SNP rs145204276 subgroups, and thus may cause statistical bias. Besides, we only extracted and analyzed the DNA of *GAS5* SNPs, no circulating RNA or plasma was retained, and both the biochemical markers and the expression of the *GAS5* long non-coding RNA itself cannot be quantified or analyzed. The absence of biochemical markers and RNA analyses could impair the overall findings of the current study significantly. Moreover, the diagnosis of DR was made by different ophthalmologists and the clinical judgment from each physician may be different. Nevertheless, all the ophthalmologists used the same criteria to diagnose DR, NPDR, and PDR which are shown in the method section in our study. Consequently, the definition of DR among them should be the same.

## 5. Conclusions

In conclusion, the *GAS5* SNP rs145204276 Del/Del variant is more prevalent in the DR population especially for those with NPDR. Furthermore, this variant is correlated with a shorter disease duration of DM in DR patients. Accordingly, genetic analysis may be recommended for those with early-onset DR whose blood sugar level is not abnormally high. Further prospective large studies to evaluate whether *GAS5* SNPs can alter the treatment outcomes of DR are necessary.

## Figures and Tables

**Table 1:** Clinical and laboratory characteristics of patients with and without diabetic retinopathy.

Variable	Non-DR Group (N = 449)	DR Group (N = 273)	*p* Value
Age (years)	60.20 ± 11.24	62.57 ± 10.77	0.005 *
Male gender [*n* (%)]	237 (52.8%)	151 (55.3%)	0.509
Duration of DM (years)	9.43 ± 7.04	12.02 ± 7.99	<0.001 *
HbA1c [% (mmol/mol)]	6.96 ± 0.99	7.59 ± 1.42	<0.001 *
Insulin treatment [*n* (%)]	105 (23.4%)	127 (46.5%)	<0.001 *
Body mass index [kg/m^2^]	26.16 ± 4.32	25.98 ± 4.31	0.588
Systolic blood pressure [mmHg]	135.34 ± 15.30	137.29 ± 17.33	0.117
Diastolic blood pressure [mm Hg]	76.36 ± 11.28	75.75 ± 11.48	0.483
Serum creatinine [mg/dL]	0.89 ± 0.35	1.55 ± 1.85	<0.001 *
Glomerular filtration rate [mL/min]	78.41 ± 27.76	62.91 ± 34.30	<0.001 *
Total cholesterol [mmol/L]	160.66 ± 43.15	165.456 ± 47.85	0.173
HDL cholesterol [μmol/L]	46.30 ± 12.66	43.77 ± 13.52	0.013 *
LDL cholesterol [μmol/L]	86.70 ± 28.31	86.71 ± 32.88	0.997
Triglycerides, [μmol/L]	140.62 ± 165.76	155.90 ± 116.83	0.193

N: number; DR: diabetic retinopathy; DM: diabetes mellitus; HbA1c: glycated hemoglobin. * denotes a significant difference between the two groups

**Table 2:** The adjusted odds ratio and 95% confidence intervals of diabetic retinopathy associated with *GAS5* genotypic frequencies.

Variable	Non-DR Group (N = 449)	DR Group (N = 273)	AOR (95% CI)	*p* Value
**rs145204276**				
Ins/Ins	206 (45.9%)	107 (39.2%)	1.000 (reference)	
Ins/Del	204 (45.4%)	129 (47.3%)	1.228 (0.850–1.773)	0.274
Del/Del	39 (8.7%)	37 (13.6%)	2.487 (1.424–4.344)	0.001 *
Ins/Del + Del/Del	243 (54.1%)	166 (60.8%)	1.189 (0.999–1.415)	0.051
**rs55829688**				
TT	212 (47.2%)	125 (45.8%)	1.000 (reference)	
TC	186 (41.4%)	119 (43.6%)	0.994 (0.694–1.424)	0.274
CC	51 (11.4%)	29 (10.6%)	0.789 (0.439–1.416)	0.427
TC + CC	237 (52.8%)	148 (54.2%)	0.974 (0.822–1.155)	0.762

N: number; DR: diabetic retinopathy; AOR: adjusted odds ratio, estimated by multiple logistic regression models after controlling for age, the duration of DM, HbA1c, insulin treatment, serum creatinine levels, glomerular filtration rate, and HDL cholesterol levels. CI: confidence intervals. * denotes a significant difference in the distribution of polymorphism between the two groups.

**Table 3:** The adjusted odds ratio and 95% confidence intervals of non-proliferative diabetic retinopathy associated with *GAS5* genotypic frequencies.

Variable	Non-DR Group (N = 449)	NPDR Subgroup (N = 163)	AOR (95% CI)	*p* Value
**rs145204276**				
Ins/Ins	206 (45.9%)	57 (35.0%)	1.000 (reference)	
Ins/Del	204 (45.4%)	80 (49.1%)	1.350 (0.852–2.000)	0.220
Del/Del	39 (8.7%)	26 (16.0%)	2.917 (1.574–5.406)	0.001 *
Ins/Del + Del/Del	243 (54.1%)	106 (65.0%)	1.242 (1.016–1.519)	0.034 *
**rs55829688**				
TT	212 (47.2%)	70 (42.9%)	1.000 (reference)	
TC	186 (41.4%)	75 (46.0%)	1.040 (0.688–1.572)	0.853
CC	51 (11.4%)	18 (11.1%)	0.997 (0.525–1.891)	0.992
TC + CC	237 (52.8%)	93 (57.1%)	1.015 (0.835–1.234)	0.880

N: number; DR: diabetic retinopathy; NPDR: non-proliferative diabetic retinopathy. AOR: adjusted odds ratio, estimated by multiple logistic regression models after controlling for age, the duration of DM, HbA1c, insulin treatment, serum creatinine levels, glomerular filtration rate, and HDL cholesterol levels. CI: confidence intervals. * denotes a significant difference in the distribution of polymorphism between the two groups.

**Table 4:** The adjusted odds ratio and 95% confidence intervals of proliferative diabetic retinopathy associated with *GAS5* genotypic frequencies.

Variable	Non-DR Group (N = 449)	PDR Subgroup (N = 110)	AOR (95% CI)	*p* Value
**rs145204276**				
Ins/Ins	206 (45.9%)	50 (45.5%)	1.000 (reference)	
Ins/Del	204 (45.4%)	49 (44.5%)	1.086 (0.635–1.859)	0.763
Del/Del	39 (8.7%)	11 (10.0%)	1.458 (0.617–3.448)	0.391
Ins/Del + Del/Del	243 (54.1%)	60 (54.5%)	1.070 (0.829–1.382)	0.603
**rs55829688**				
TT	212 (47.2%)	55 (50.0%)	1.000 (reference)	
TC	186 (41.4%)	44 (40.0%)	0.940 (0.551–1.605)	0.822
CC	51 (11.4%)	11 (10.0%)	0.502 (0.184–1.371)	0.179
TC + CC	237 (52.8%)	55 (50.0%)	0.916 (0.710–1.181)	0.497

N: number; DR: diabetic retinopathy; PDR: proliferative diabetic retinopathy. AOR: adjusted odds ratio, estimated by multiple logistic regression models after controlling for age, the duration of DM, HbA1c, insulin treatment, serum creatinine levels, glomerular filtration rate, and HDL cholesterol levels. CI: confidence intervals.

**Table 5:** Clinical characteristics of diabetic retinopathy patients according to *GAS5* rs145204276 genotypes.

Variable	*GAS5* rs145204276		
	Ins/Ins (N = 107)	Ins/Del (N = 129)	Del/Del (N = 37)
Duration of DM (years)	13.51 ± 8.36	11.51 ± 8.05	9.46 ± 5.64 ^a,b^
HbA1c [% (mmol/mol)]	7.73 ± 1.51	7.56 ± 1.34	7.30 ± 1.43
Serum creatinine [mg/dL]	1.75 ± 2.40	1.51 ± 1.45	1.13 ± 1.06
Glomerular filtration rate [mL/min]	60.70 ± 33.31	62.61 ± 35.98	70.08 ± 31.07
Total cholesterol [mmol/L]	161.02 ± 44.56	168.54 ± 50.58	167.43 ± 47.70
HDL cholesterol [μmol/L]	43.82 ± 13.00	43.67 ± 13.80	43.99 ± 14.34
LDL cholesterol [μmol/L]	83.70 ± 29.82	90.18 ± 35.35	83.60 ± 32.21
Triglycerides, [μmol/L]	147.19 ± 101.52	155.57 ± 117.50	180.51 ± 149.17

*GAS5*: growth arrest-specific 5; N: number; DM: diabetes mellitus; HbA1c: glycated hemoglobin; ^a^ ANOVA analysis with Tukey’s a posteriori comparison was used. F = 4.099, *p* = 0.018. ^b^ Significantly different, *p* = 0.021, when compared to the Ins/Ins group.

## Data Availability

The data presented in this study are available on request from the corresponding author.
